# Nafion Modified Titanium Nitride pH Sensor for Future Biomedical Applications

**DOI:** 10.3390/s23020699

**Published:** 2023-01-07

**Authors:** Shimrith Paul Shylendra, Magdalena Wajrak, Kamal Alameh, James Jin Kang

**Affiliations:** 1 School of Science, Edith Cowan University, Joondalup, WA 6027, Australia; 2 International College, National Taiwan University, Taipei 10617, Taiwan

**Keywords:** pH sensing, high redox samples, Titanium Nitride (TiN), Nafion, smart sensors, medical applications

## Abstract

pH sensors are increasingly being utilized in the biomedical field and have been implicated in health applications that aim to improve the monitoring and treatment of patients. In this work, a previously developed Titanium Nitride (TiN) solid-state pH sensor is further enhanced, with the potential to be used for pH regulation inside the human body and for other biomedical, industrial, and environmental applications. One of the main limitations of existing solid-state pH sensors is their reduced performance in high redox mediums. The potential shift E^0^ value of the previously developed TiN pH electrode in the presence of oxidizing or reducing agents is 30 mV. To minimize this redox shift, a Nafion-modified TiN electrode was developed, tested, and evaluated in various mediums. The Nafion-modified electrode has been shown to shift the E^0^ value by only 2 mV, providing increased accuracy in highly redox samples while maintaining acceptable reaction times. Overcoming the redox interference for pH measurement enables several advantages of the Nafion-modified TiN electrode over the standard pH glass electrode, implicating its use in medical diagnosis, real-time health monitoring, and further development of miniaturized smart sensors.

## 1. Introduction

pH monitoring and regulation is crucial in both biomedical and non-biomedical applications. In biological systems, pH maintenance is important to sustaining health and physiological equilibrium, and a disturbance in pH levels may reflect an underlying dysfunction or pathological process [1]. Certain examples of pH measurement include its use in detection of glioblastomas (type of brain tumor), monitoring of ischemic episodes, and testing of bodily fluids, such as urine, saliva, sweat, and blood [2]. Non-medical applications include the use of pH monitoring in chemical industries, agriculture, water management, food safety control, and the cosmetics industry.

In a specific application of pH in medical science, for instance, when labelling Prostate-specific membrane antigen (PSMA) peptide with lutetium-177 (Lu-177) or actinium-225 (Ac-225) in the radiopharmaceutical treatment of prostate cancer, the pH of the radioactive lutetium or actinium must be at pH = 9 to allow for complete binding of the peptide. Therefore, it is necessary to monitor the pH throughout the chemical reaction [3,4]. At present, a universal pH paper is used to monitor the pH of the Lu-177 and Ac-225 solutions, as glass pH electrodes cannot be placed into a 1 mL solution of radioactive substance. However, universal pH paper only provides a rough estimate of the pH and is prone to external factors, such as user error and lighting of test conditions due to the inability of the user to recognize color changes in the pH paper [5]. Consequently, a cheap, accurate, and disposable pH solid-state electrode would be ideal for this situation. Another example of the importance of being able to accurately monitor pH is in the development of Covid-19 vaccines. Throughout the manufacturing process of the Covid-19 vaccines, the pH is required to be maintained between 7.1 and 7.2 and, therefore, requires constant and accurate monitoring. Solid-state electrodes such as TiN can be ideal for such applications [6]. Although glass electrodes are the industry standard for measuring pH, they are not appropriate for all applications due to their fragility and size, limiting their use for medical purposes [7].

The pH sensors developed in this paper are evaluated in the various mediums and matrices for possible future medical, industrial, and environmental applications [8,9]. The two fundamental limits addressed in this work are the application of matrix and the microscopic size [10]. A smart sensor can be used to measure physiological characteristics, such as the acidity of drinking water at remote locations or in conflict zones, to overcome these restrictions. Soldiers in conflict zones will be able to keep track of their health conditions and issues with the use of convenient and accessible pH testing equipment. The most important discoveries and contributions of this work are that (1) TiN thin films have the potential for the development of miniaturized pH sensors and (2) Nafion-modified TiN electrodes have potential to be used for many new pH sensing applications.

Metal oxide pH electrodes were once an alternative to glass pH electrodes [11,12,13,14]. IrO_2_-based pH electrodes and their less popular RuO_2_-based counterparts have both been the subject of extensive research [12]. Metal oxides’ primary flaw is their redox sensitivity [15]. This redox sensitivity has a negative impact on the electrode potential in the neutral region and, consequently, limits the use of these materials as sensors [16]. The disruption of reducing or oxidizing species causes this constraint to be most noticeable in potentiometric metal oxide electrodes. Fog and Buck [17] claim that RuO_2_ exhibits significant changes in redox agents. However, they also state that other iron oxides, such as IrO_2_, do not retain a Nernstian pH response above half the pH range when there are oxidizing or reducing agents [6,9]. It is interesting to note that this claim was only superficially addressed in their 1984 book and has not been adequately explored in the literature. To address this issue, other researchers investigated the use of Nafion to modify pH electrodes, such as an IrO_2_ sensor [18], and they were successful in minimizing electrode potential shift in the neutral pH range. It was demonstrated in previously published work that adding protective layers of Ta_2_O_5_ [13] and Nafion [19,20] to a RuO_2_ pH electrode lowers the redox interference and gives more accurate pH values of some samples, such as beer [11]. These modified electrodes, however, were still ineffective for samples such as wine and fresh citrus juice [11]. In this study, not only has it been possible to develop a sensor that is highly accurate in samples, but also has an improvement with a small potential shift of 2 mV, when compared to a RuO_2_ sensor, which has a shift of 300 mV, and an unmodified TiN sensor with a shift of 30 mV in reducing conditions [21,22]. Having overcome the redox interference in metal nitride pH sensors now allows for the possibility of replacing the glass electrode in applications where the size of the electrode and robustness are crucial, and where there is a need for low production costs [23,24,25,26,27].

This work is a follow up study from previous studies conducted on metal oxides and nitrides, i.e., RuO_2_ [7,11], RuN [23], Br-C-N [28], SiN [29], TiN [21,30], ZiN, and HfN [31], as summarized in Table 1 below.

TiN is proven to be a biocompatible material for medical and chemical applications [33,34]. However, the reaction of the metal-oxide or metal-nitride film with the testing medium remains a gap in the literature. Interference of pH material with high-redox species, such as ascorbic acid and orange juice, is a major disadvantage with oxidation surfaces used as pH sensors [35]. To address this gap, TiN was modified with Nafion to protect the material from interacting with other species in the sample, which in turn affects the sensitivity of the sensor [36,37]. Overcoming this issue paves the path for the future development of a robust and reliable pH sensor for chemical and biological applications [38,39]. Experiments, analysis, and applications of the TiN sensor with Nafion modification are presented in the following sections.

## 2. Materials and Methods

**Figure 1:**
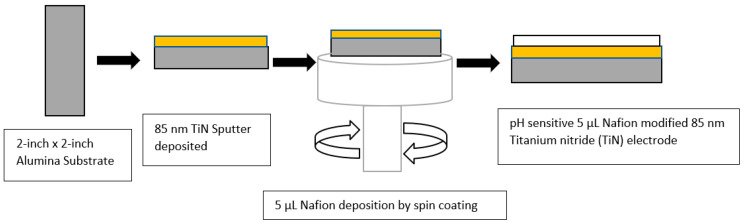
Schematic representation of 85 nm TiN electrode modified with Nafion.

Working electrodes made of TiN that are pH sensitive were fabricated by sputtering TiN over an Al_2_O_3_ substrate that was 0.5 mm thick. A TiN target (99.95% purity) with 110 W of sputter power was used to deposit TiN, utilizing radio frequency magnetron sputtering (RFMS) at room temperature. Various gas pressures in the gas chamber and argon: oxygen gas ratios were tested, as described in previous publications [14,21]. In Figure 1, the TiN-sputtered layer created a vividly colored gold coating on a glass substrate [22]. A total of 5 µL of 5% Nafion 117 in a mixture of aliphatic alcohol and water from Sigma was spin coated on top of 85 nm TiN. It was then annealed for 25 min at 150 °C.

### 2.1. pH Measurements

Using a high impedance voltmeter (Agilent, Santa Clara, CA, USA), potentiometric measurements were recorded between the TiN + Nafion working electrode and a commercial Ag|AgCl|KCl glass, double-junction, reference electrode (Sigma-Aldrich, St. Louis, MO, USA). At 22 °C, commercial buffer solutions (Rowe Scientific, Minto, Australia) were used for the analysis. Each measurement was completed in five minutes. The movement of the potential in pH 7 buffer over the duration of the analysis time is given by the drift numbers, with error bars indicating the 95% confidence interval [10,23,24]. For each measurement, the last 30 s of analysis were averaged and used to calculate sensitivity, E°, and hysteresis.

The selective identification of H^+^ ions present in the examined solution is necessary for potentiometric pH determination. A measurement device and an electrochemical cell make up the typical potentiometric configuration (potentiometer, voltmeter, multimeter, etc.). A pH-sensitive sensor electrode and a reference electrode make up the electrochemical cell (usually silver chloride electrode). Electromotive force is an electrochemical cell’s electrical characteristic (Emf). The difference in electrode potentials (E) of the two half-reactions occurring at the sensing and reference electrodes is used to determine the cell’s Emf. The Emf of the cell is typically equal to the potential of the sensing electrode, and the reference electrode is grounded, with its potential regarded as equal to zero. The half-reaction occurs in response to detecting, where E^0^ is the reference voltage,

E = E^0^ − R·T n·F·ln [Red] [Ox] (1)

where R is the universal gas constant, 8.314 J/Kmol; T is the temperature, K; and n is the quantity of electrodes involved in the redox reaction. The activities of the reduced and oxidized versions of the electrode material, respectively, are [Red] and [Ox], measured in mol/L, and F is the Faraday constant, which is equal to 96,485 C/mol.

The standard potential is a measurement of the equilibrium individual potential of the reversible electrode in the standard condition (1 mol/L concentration, 1 atm pressure, and 25 °C temperature). The following simplified equation, which was proposed by [40,41], may be used to explain the pH-sensing mechanism for the TiN electrode:

Ti(III) + e^−^ + [H]^+^ ↔ Ti(II) (2)

The Nernst equation for this process takes the following form:

E = E^0^ Ti(III)/Ti(II) − R·T n·F·ln [Ti(II)] [Ti(III)]·[H^+^] (3)

where [Ti(III)], [Ti(II)], and [H^+^] are activities. Equation (3) has the following form at room temperature (T = 22 °C), considering that the values of metal activities in solids are close to 1:

[Ti(III)/Ti(II)] 0.0583 log [H^+^] + E = E^0^ (4)

Electrode sensitivity, or the theoretical Nernst response at 22 °C, is defined as the value of 58.3 mV. When n = 1 in Equation (3), all pH-sensitive electrodes at the specified temperature should have the same sensitivity value; however, the theoretical response, in practice, will deviate from this value [33,34].

Prior to the initial measurement, all electrodes underwent a conditioning routine that involved soaking them in distilled water for 24 h to hydrate pH-sensitive surfaces. The sensitivity of the manufactured electrodes was assessed by detecting the electrochemical cell’s electromagnetic field (the potential difference between the reference electrode and the fabricated pH-sensitive electrode) as a function of pH. For that, electrodes were submerged into buffer solutions of pH range from 1 to 14. Every 10 s, data points were obtained while the Emf was recorded for 5 min. The average value of the previous 10 data points was used to calculate the Emf at that pH. By charting the electrode potential as a function of pH and figuring out the equation expressing this dependency, the electrode sensitivity, E^0^, and linearity of the response were found, using the least-squares method. E^0^ was estimated as the potential at pH = 0 by extrapolating the data, and the linearity of the electrode’s response to pH change was calculated as the correlation coefficient. Electrode sensitivity was computed as the slope of the linear equation.

### 2.2. Response Time, Drift Rate, and Hysteresis

The time required for the electrode potential to reach 90% of the stable value was used to calculate the response time. Electrodes were left in distilled water overnight to assess the drift of electrode response in time. The drift rate (in mV/h) was calculated using the slope of the line-of-best-fit method. The created electrodes were subjected to a variety of pH buffers to evaluate the hysteresis, or memory effect, of an electrode. The pH of the electrodes was first changed from 1.1 to 4.1 to 7.0 to 10.0 (acidic to basic), and then the pH was changed in the other direction. After dipping the electrode into a fresh buffer solution, the electrode response was monitored for three minutes. A solution of cleaned electrodes was washed with distilled water and dried with a pressure gun after each measurement.

### 2.3. Measurements of Real Samples

The fabricated TiN + Nafion electrodes were used to measure the pH values of different types of samples: cola, beer, red wine, white wine, orange juice, fresh lemon juice, and iced tea. The glass commercial electrode was used to test all these samples as a reference to the TiN pH sensor.

## 3. Results and Analysis

This section records the detailed step-by-step fabrication process of TiN + Nafion electrodes. The key performance indicators of pH sensors, such as sensitivity, drift, and hysteresis, are explained and reported in this section of the paper. Finally, the TiN + Nafion electrode was applied in real high-redox sample varieties to test the performance and validate the role of Nafion modification to TiN pH sensors.

### 3.1. Deposition Parameters

To find the best manufacturing conditions for all-TiN pH electrodes, the TiN sputter deposition gas pressure and Ar: N_2_ gas ratio were briefly investigated. Two alternative substrates were tested: polished Al_2_O_3_ and glass at Ar: N_2_ gas ratio of 1:9. By soaking samples in a deionized water ultrasonic bath for 10 minutes and putting them through a peel adhesion test using polyimide adhesive tape, samples were examined for adhesion. When the polyamide was evaluated for pH characteristics, it demonstrated greater adhesion. Using pH 2, 4, 7, 10, and 12 buffers, the pH sensitivity of the samples was assessed. All the TiN electrodes showed Nernst response, although electrodes with thicker than 5 µL of Nafion showed a significant amount of drift, as shown in Table 2.

Based on these results, the best electrode characteristics for TiN are when sensitivity is close to Nernstian and hysteresis and drift are both low. It was found that an 85 nm TiN electrode exhibited Nernstian sensitivity of 56.4 mV/pH, 2.3 mV hysteresis, and had the lowest drift of 4.6 mV/h when 5 mL of Nafion was applied. Hence, this thickness of Nafion was selected to spin coat on TiN to determine the sensing properties of the electrode.

In addition, this electrode, when left in pH 7 buffer for 4 months, maintained its sensitivity. This is a significant finding because other metal-oxide solid-state electrodes demonstrate signs of degradation in less than 4 months [20]. This is likely due to the specific construction of the TiN electrode, where the effects from electrical contact materials, such as carbon, platinum, or gold, are eliminated.

### 3.2. Sensing Properties

**Figure 2:**
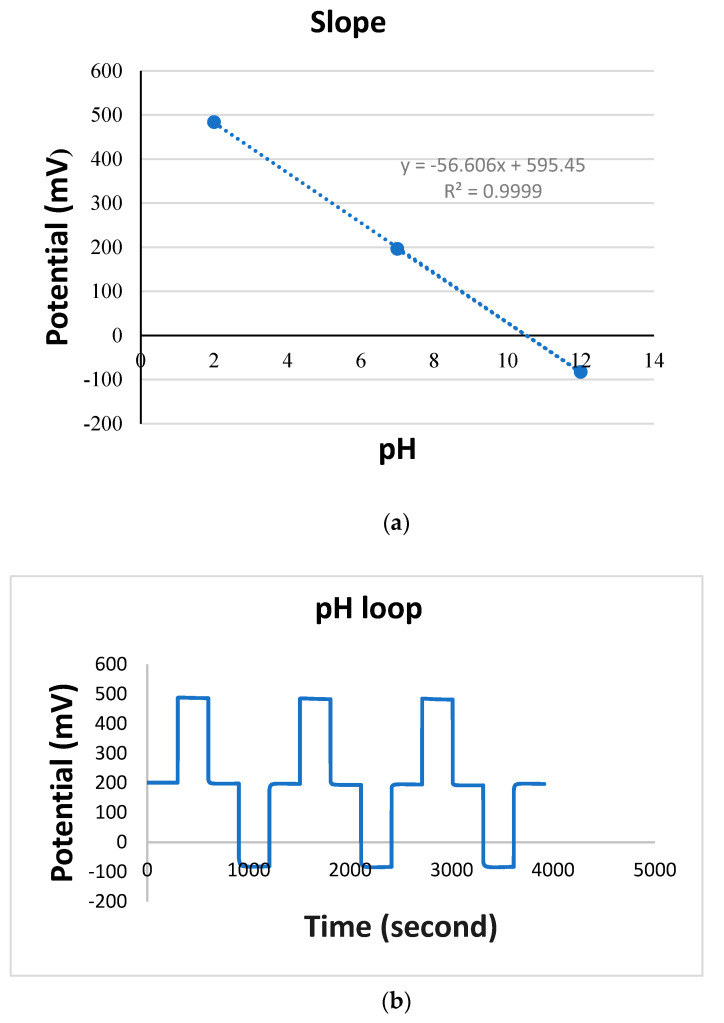
(**a**) Linear calibration plot for pH sensor from pH 2 to 12, (**b**) pH loop cycled 7-2-7-12-7 three times, using potential data versus time for TiN working electrode in pH 7 buffer vs. glass reference electrode.

The TiN + Nafion pH sensor was examined by looping pH from 2 to 12, as shown in Figure 2a. The developed sensor shows Nernstian sensitivity (−56.6 mV/pH) and a reaction time of less than 30 s, which is consistent with previous reports of pH sensors employing this type of working electrode [14,15]. As seen in Figure 2b, the pH response is linear (R^2^ = 0.9999) and reproducible, as summarized in Table 2. The R square value is the correlation coefficient, which indicates the excellent linearity of the slope.

The specific construction of the TiN + Nafion sensor reported here has resulted in several advantages compared to the solid-state electrodes previously reported [14]. The biggest advantage is that almost all the sputtered material is used, hence, there is no wastage, and this also allows for uniform production of bulk number of electrodes.

**Figure 3:**
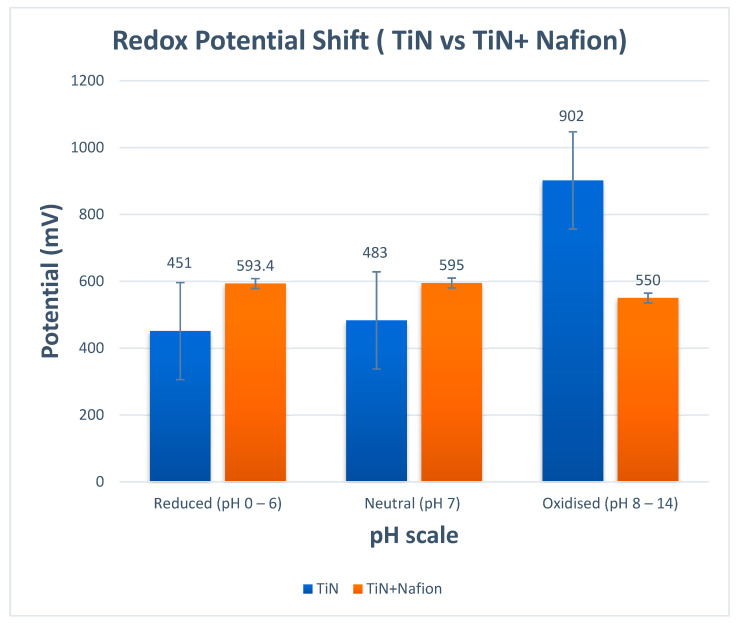
Potential versus different matrices for TiN and TiN + Nafion working electrode.

The 85 nm-thick TiN + Nafion pH-sensitive electrode was deposited at a pressure of 2 mT and a gas ratio of 1:9 Ar:N_2_ to study the effects of redox agents. Then, 1 mM ascorbic acid or MnO_4_ were added to buffer solutions. As shown in Figure 3, the electrode was calibrated at pH 7 (with a redox agent) for 15 min before pH was looped 3 times from 7-2-7-12. As shown in Figure 3, in neutral and reducing conditions, the shift in the redox potential has now been significantly minimized (2 mV) as an expected outcome. This is exactly the outcome we were hoping to achieve. Unfortunately, when pH measurements were performed in buffer solutions with oxidizing redox agents, there was still potential shift close to 45 mV. However, the good news is that the shift was stable over that range of pH values and, more importantly, the shift was smaller than for TiN without the Nafion protection.

Careful examination of the data in Figure 3 confirms that the TiN + Nafion electrode’s behavior in different redox solutions corresponded to a change in the E* value while maintaining Nernstian sensitivity, as shown in Figure 2. When exposed to a new test solution, the potential for each pH measurement quickly changed and equilibrated within 30 s, after which the observed potential started to drift. The pH sensitivity of the electrode can be determined using the difference between the final reading from the previous measurement and the 30 s equilibrated value, as shown in Figure 2, assuming that this drift is caused by a shift in E* rather than sensitivity.

### 3.3. Drift Rate

TiN + Nafion electrodes were subjected to various pH conditions, ranging from 2 to 12 for varied reaction periods (15, 60, 120, or 240 min), to investigate the drift effect. The drift of the electrodes obtained in pH = 2 and pH = 12 solutions showed an apparent decrease from 36.18 to 6.4 mV/h and 22.53 to 5.16 mV/h, respectively, as shown in Table 3, with an increase in reaction time from 15 to 240 min. The TiN electrode drifted considerably more than we expected, which may be attributed to the high resistance and inherent characteristics, such as the TiO_2_ sheets [8]. Yusof [9] reported that TiO_2_-sensing membrane fabricated using the RF sputtering method has a drift of 4.41 mV/h. However, when TiO_2_ film was doped with ruthenium metallic ions, using a co-sputtering system to decrease the resistivity and increase its carrier mobility, that resulted in a very low drift effect of only 1.67 mV/h. In conclusion, the TiN + Nafion electrode exhibits good stability with increased reaction time, which means that the sensor reaches its equilibrium when reaction time is increased, and, hence, acceptable drift of 7 mV/h (average across pH 2 to 12) is achieved. The longevity of the sensor beyond 6 months remains a further area of study.

### 3.4. Hysteresis

Previous work by Fog and Buck [17] showed the hysteresis width of the TiN pH sensors to be 30 mV in the cycle of 2-12-2. The hysteresis width of TiN pH electrodes has only been briefly described in previous research papers [30,32,39]. The TiN electrodes manufactured here have a comparatively high hysteresis. For 5 µL of Nafion, 60 min hysteresis was 17.6 mV to 19.3 mV, and for 120 min, hysteresis was 8.9 mV to 11.4 mV, and for 10 µL, 60 min hysteresis was 10 mV to 10.5 mV, and for 120 min, it was 7.3 mV to 9.5 mV [17]. This is higher when compared to other metal-oxide-based pH-sensitive electrodes. According to previous research [39], a magnetron-sputter-fabricated RuO_2_ pH sensor has a hysteresis of 6.4 mV in the loop of 7-4-7-10-7 and 5.1 mV in the loop of 7-10-7-4-7, and for electrodeposited iridium oxide, the pH sensor ranges from 0.5 to 1.5 mV.

With repeatability based on the characterization studies, the ideal manufacturing parameters for the super-hydrophilic TiN pH electrode were determined to be 9.29 mV/h and 120 min. In addition, these conditions displayed the highest sensitivity of 54.13 mV/pH, the fastest response of 18.1 s in the pH range of 4 to 12, a tolerable drift of 9.29 mV/h, and the widest hysteresis at 11.4 mV. A new series of electrodes was then created to test the reproducibility of these electrodes. For each pH level, the new series of electrodes displayed steady and consistent response potentials and showed a comparable Nernstian response, with a sensitivity of 54.55 and 56.58 mV/pH. Thus, the discrepancy in the sensitivity for the various manufactured electrodes was less than 1.95 mV/pH, indicating very good repeatability when compared with previously reported electrodes [38].

### 3.5. Real Samples Application

To determine the performance of the TiN + Nafion electrode in strong reducing and oxidizing matrices, various real samples were tested. Consequently, an 85 nm TiN + Nafion electrode (2 m Torr, 1:9 Ar:N_2_) was tested in solutions such as coke, beer, white wine, red wine, iced tea, orange juice, and fresh lemon juice. Firstly, the sensor was rinsed with deionized water and equilibrated in pH 4 or 7 buffer standard for 90 s in order to obtain a standard value for single-point calibration of the sensor’s E* value [7]. Calculations were performed using the sensitivity of −56.6 mV/pH, as determined previously. Sample pH values were calculated using the pH sample equation [11]. The equation and buffer standard were chosen to minimize the pH difference between the standard and sample tests to provide the highest accuracy of the single-point calibration [7].

**Figure 4:**
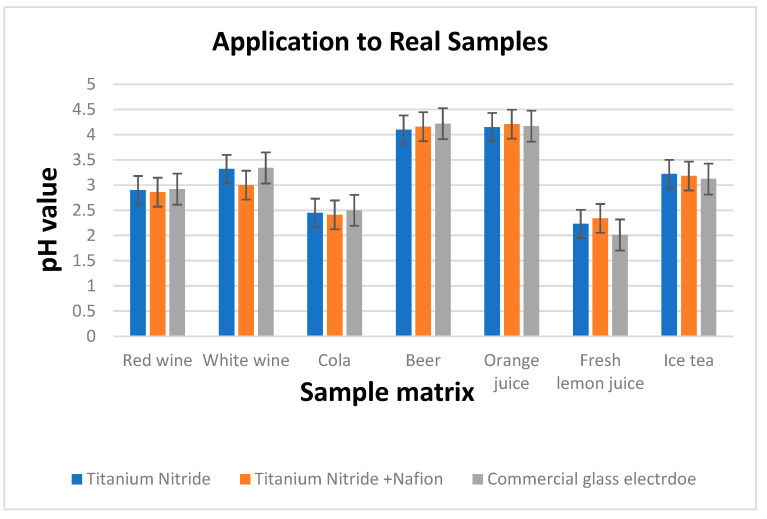
pH values determined using 85 nm TiN electrode with the developed TiN + Nafion and using a commercial glass pH sensor for seven samples.

Figure 4 compares the pH readings obtained using a commercial glass pH sensor and an 85 nm TiN electrode to those obtained using the TiN + Nafion. The results shown in Figure 4 illustrate that there is excellent consistency between the pH values obtained using this measurement methodology and those obtained using a commercial glass pH sensor (EU Tech). These experimental findings show that a variety of sample matrices can be used with TiN + Nafion electrodes. As mentioned earlier, the RuO_2_ sensor exhibited ±300 mV shifts in potential and instability in many of the samples, resulting in a poor performance. The TiN + Nafion sensor developed here shows improved performance, with pH values on average within 0.25 pH units of the commercial glass electrode. In addition, the TiN + Nafion sensor outperforms the differential pH sensor developed by previous researchers because it exhibits significantly less potential shift of 2.1 mV (595 mV − 593 mV = 2 mV), as shown in Figure 3.

Accurate pH measurement of the samples, such as white wine and fresh citrus juice, using solid-state electrodes has not been feasible before due to the presence of ascorbic acid and other redox active compounds, such as preservatives. These types of samples cause large shifts in potential due to the oxidization/reduction of the working electrode [7]. The result of our work demonstrates that a differential pH sensor based on TiN film with Nafion coating can function as a reliable pH sensor in matrices that have been previously problematic [42].

## 4. Conclusions

An all-solid-state potentiometric pH sensor was successfully developed, which employs a thin-film sputter-deposited TiN + Nafion (spin coated) working electrode and an Ag|AgCl|KCl glass electrode as a reference. A durable pH-sensitive electrode has been manufactured entirely from sputter-deposited TiN using a TiN sputter target at 2 m Torr gas chamber pressure and with 1:9 Ar:O_2_ gas ratio. It has been demonstrated here that the TiN/Nafion sensor can be used to overcome redox interference and can give accurate (precision of ±0.25 pH units) pH values in samples, such as wine and fresh citrus juice, where metal-oxide type pH sensors were previously unable to accurately measure. Experimental results have shown that the sensor we developed not only exhibits a Nerstian linear pH response (−56.6 mV/pH, R^2^ = 0.9999), but also has excellent reproducibility (hysteresis < 2 mV) without the need of sample calibration, unlike previous sensors. Additionally, only with a single-point calibration measurement protocol, the sensor can attain a moderate level of accuracy (±0.25 pH) when applied to cola, milk, yogurt, and beer samples.

Experimental results have demonstrated that, for orange juice and wine samples, with the TiN, the electrode potential shift is reduced and an improved accuracy of ±0.25 pH can be attained. Manufacturing of TiN is considerably cheaper than RuO_2_ and Pt and Au electrodes, which are also robust, accurate, and cheaper than standard glass electrodes. Additionally, the measurement protocol is well suited for low-throughput analysis, particularly by unskilled operators, which may be utilized for domestic applications. Further miniaturized development of this electrode and validation with biological samples could see this electrode be applied in the medical field, especially in situations where current glass electrodes cannot be used due to fragility and size constraints. For example, gastrointestinal reflux disease requires pH monitoring and currently diagnosis is conducted using endoscopy. However, developing this work into a nano capsule can help retrieve data by wireless signaling from the sensor without any medical intervention to a smart device such as smartphones, which can transmit the data to a server in cloud networks for physicians or health service providers to access for their services.

## Figures and Tables

**Table 1:** Comparison of current work on metal nitride-based pH sensors applied in redox matrix to previous work from the literature. * No conditioning protocol required for this sensor as compared to the RuO_2_ sensor.

Application Matrix	pH Sensitive Material	Fabrication Method	Sensitivity (mV/h)	pH Range	Reference
Redox matrix	RuO_2_	RF sputtering	−56.6	2–12	[7]
Biological and environmental application	RuN	Magnetron sputtering	−58.3	1–12	[23]
Phosphate buffer solution	Br-C-N	Dual gun sputtering	−46	1–13	[28]
Aquaculture	SiN	ISFET package	−53.6	4–10	[29]
Chemical applications	InN	ISFET	−58.2	2–12	[32]
Chemical application	IrO_2_ + Nafion	Cryogenic sputtering	−60.2	2–12	[21]
Fresh Orange juice	TiN	RF Magnetron sputtering	−59.1	2–12	[21] previous work
Common drinks with redox species	TiN + Nafion	RF Magnetron sputtering	−56.6	2–12	* This work

**Table 2:** Summary of the pH-sensing performance for TiN electrodes with various Nafion thickness ranging from 5 to 25 μL thickness, with TiN deposited using 2 m Torr pressure and 1:9 Ar: N_2_ gas ratio.

Resistivity (ohm)	Sputter Target	Gas Ratio (Ar:N_2_)	Sputter Pressure (mTorr)	Nafion Thickness (µL)	Sensitivity (mV/pH)	Hysteresis (mV)	R^2^	Drift (mV/h)
2.6	Ti	9:1	2	5	−56.4 ± 1.2	2.3 ± 1.2	0.9997	4.6 ± 1.2
4.1	Ti	9:1	2	10	−59.3 ± 3.2	84.7 ± 3.4	0.9341	78.48 ± 2.5
4.8	Ti	9:1	2	15	−56.2 ± 2.8	52.63 ± 1.2	0.9818	165.4 ± 6.7
4.9	Ti	9:1	2	20	−53.4 ± 4.2	55.17 ± 4.7	0.9698	210.56 ± 2.7
5.6	Ti	9:1	2	25	−53.1 ± 1.2	71.30 ± 7.8	0.9519	222.84 ± 3.8

**Table 3:** Drift of TiN electrodes treated using different pH parameters.

pH	Reaction Time (min)			
	15	60	120	240
2	36.16 mV/h	15.22 mV/h	9.32 mV/h	6.20 mV/h
4	18.26 mV/h	12.54 mV/h	10.33 mV/h	7.88 mV/h
7	15.33 mV/h	12.45 mV/h	9.44 mV/h	4.22 mV/h
10	42.56 mV/h	34.88 mV/h	19.73 mV/h	12.45 mV/h
12	22.43 mV/h	9.36 mV/h	7.11 mV/h	4.26 mV/h

## Data Availability

The data presented in this study are available on request from the first author. The data are not publicly available as further study will be carried out using the same data.
